# Transcriptome analysis reveals rootstock-driven effects on growth and photosynthesis in *Camellia chekiangoleosa*: A phenotypic and biochemical perspective

**DOI:** 10.1371/journal.pone.0331313

**Published:** 2025-09-03

**Authors:** Zexin Chen, Linqing Cao, Chuansong Chen, Qiuping Zhong, Tieding He, Jinfeng Wang, Youcheng Zhou, Yuling Zou, Xiaoning Ge

**Affiliations:** 1 Experimental Center of Subtropical Forestry, Chinese Academy of Forestry, Fenyi, China; 2 Institute of Forest Resource Information Techniques, Chinese Academy of Forestry, Beijing, China; Institute for Horticultural Plants, China Agricultural University, CHINA

## Abstract

*Camellia chekiangoleosa* is a significant oil-bearing tree species, known for its high oleic acid content and shorter reproductive cycle compared to traditional oil-tea plants. However, there are few studies on the molecular mechanism and compatibility of the interaction between oil-*Camellia* scion and rootstock, which poses certain challenges to the cultivation and promotion of oil-*Camellia*. This study systematically evaluates the effects of hetero-grafting *Camellia chekiangoleosa* scions onto divergent rootstocks (*Camellia chekiangoleosa*, *Camellia oleifera*, and *Camellia yuhsienensis*). Then the research investigates how rootstock selection alters scion growth and development through phenotypic, biochemical, and transcriptomic analyses. Our findings reveal that the combination of *C. oleifera* scion grafted onto *C. yuhsienensis* suppresses auxin (IAA) and cytokinin (ZR) levels while elevating abscisic acid (ABA). Transcriptomic analysis identified that the *PYL1*, *AMY*, and *INV1* screened by transcriptome data were mainly enriched in starch and sucrose metabolic pathways and plant hormone signal transduction, which collectively prioritize carbon allocation toward growth over storage. Meanwhile, hetero-grafting improved photosynthetic capacity by upregulating light-harvesting complex (*LHC*) genes and carotenoid biosynthesis enzymes (*ZEP*), optimizing light energy conversion and photoprotection. These findings provide novel insights into the molecular mechanisms underlying rootstock-scion interactions in oil-*Camellia*, bridging a critical knowledge gap in this economically important genus.

## Introduction

Oil-*Camellia*, a member of the *Theaceae* family, has been cultivated in China for over 2,000 years for its oil-rich seeds, which possess edible, medicinal, and healthcare value [1]. This species is mainly distributed across Zhejiang, Jiangxi, Hunan, Guizhou, Guangxi, and Henan provinces. Another species, *Camellia chekiangoleosa* (Cc), native to the mountainous regions of Jiangxi, Zhejiang, and northern Fujian in southern China, stands out for its short fruit ripening period and seeds with high oil content [2]. The oil extracted from *C. chekiangoleosa* is rich in unsaturated fatty acids, making it superior quality [3]. However, despite its agronomic and economic potential, *C. chekiangoleosa* faces specific cultivation challenges, including limited genetic diversity in rootstock compatibility and insufficient understanding of molecular mechanisms underlying rootstock-driven improvements in oil quality and stress resilience. Additionally, this species holds ornamental value, playing an essential role in landscaping [4]. Alongside *Camellia Oleifera* (Co), *Camellia Chekiangoleosa* has been extensively cultivated in southern China for millennia [5]. Additionally, *Camellia yuhsienensis* (Cy), another valuable oil tea species, is widely used for both ornamental and oil purposes due to its strong floral fragrance, high oil quality, and resistance to diseases [6,7].

Grafting, a common horticultural technique for woody plants, is particularly critical for *Camellia* species like *C. chekiangoleosa*, where the promotion of excellent asexual lines of oil-*Camellia* is carried out by using the bud rootstock grafting technology in actual production, which can make full use of the advantages of the rootstock, improve the quality of the scion, shorten the growth cycle, and increase yield and economic benefits [8–10]. Notably, Rootstock selection directly impacts oil yield, disease resistance, and adaptation to heterogeneous environments [11]. While prior studies in oil-*Camellia* have established grafting as a tool to enhance scion vigor and stress tolerance [9,12], the molecular and physiological interplay between *C. chekiangoleosa* scions and divergent rootstocks remains poorly characterized. However, *C. chekiangoleosa*’s rapid fruit maturation and distinct fatty acid profile necessitate tailored rootstock-scion combinations to optimize resource allocation without compromising oil quality. Thereby, in the context of oil tea cultivation, breeding *C. chekiangoleosa* with high oleic acid content and stable yields requires rootstocks that harmonize with its unique phenology and metabolic demands.Modulating the growth-defense trade-off in *C. chekiangoleosa* requires understanding rootstock-driven hormone signaling and carbohydrate partitioning.

Selecting an appropriate rootstock can significantly optimize the phenotype of grafted plants, through mechanisms that are species- and genotype-dependent. The influence of the rootstock on the phenotype of grafted plants is achieved by altering the plant’s growth morphology, plant hormones, and physiological characteristics [13,14]. In *Camellia* species, rootstock-induced transcriptional reprogramming in scions has been linked to oil biosynthesis and stress responses, but these studies predominantly focus on oil-*Camellia*, leaving *C. chekiangoleosa*’s molecular adaptability to heterografted systems unexplored [3]. Transcriptomics is increasingly used to dissect rootstock-scion interactions, yet no prior work has integrated physiological traits with multi-omics data to resolve how *C. chekiangoleosa* scions acclimate to phylogenetically divergent rootstocks like *C. oleifera* and *C. yuhsienensis* [15–17].

Here, we address these gaps by grafting *C. chekiangoleosa* scions onto rootstocks of *C. chekiangoleosa*, *C. oleifera*, and *C. yuhsienensis*—a comparative framework designed to isolate species-specific rootstock effects. We measured chlorophyll fluorescence parameters, endogenous hormone levels, and carbohydrate content in grafted seedlings, coupled with RNA-seq analysis. This approach uniquely targets *C. chekiangoleosa*’s understudied grafting biology, revealing how rootstock identity modulates its photosynthetic efficiency, hormonal homeostasis, and transcriptional networks governing oil metabolism and stress adaptation. The findings will provide a theoretical foundation for precision breeding of oil-*Camellia*, with emphasis on optimizing *C. chekiangoleosa* cultivation through rootstock-scion synergies absent in prior studies.

## Materials and methods

### Plant material and treatment

The experiment was conducted at the Experimental Center of Subtropical Forestry, Chinese Academy of Forestry (27◦33′ ~ 28◦08′N, 114◦29′ ~ 114◦51′E) in Jiangxi Province, China. Grafting combination were planted at the same location in May 2022, with standard water, fertilizer, and field management practices.

Scion materials were obtained from the vigorous annual shoots of non-grafted Cc. Two hetero-grafting and one auto-grafting combinations were constructed as follows: Cc/Cy, Cc/Co, and Cc/Cc. Seeds from non-grafted trees were screened, soaked, disinfected, and then stored in sand from December to the following May for use as rootstock materials. In May of the following year, 1-year-old shoots were collected from the same batch of non-grafted trees to serve as scion materials. These branches were required to be robust and semi-lignified. Grafting was performed using the seedling rootstock grafting technology as described by Ge [18]. Each grafting combination was replicated three times, with 100 grafted seedlings per replicate. The experimental treatments were randomized in block design, and rhizome samples 3 cm below the grafting joint were collected at 90 DAG, with 3 biological replicates each. The samples were immediately frozen in liquid nitrogen and then stored in a –80°C refrigerator for RNA-seq, RT-qPCR verification, and determination of plant hormones and carbohydrates.

### Plantlet growth and physiological parameter measurements

Five healthy individuals from each grafting combinations were randomly selected for measurement of height and basal diameter at the graft union. Afterward, the plants were harvested. Biomass was measured by weighing all samples after oven-drying at 80 °C for 48 hours to a constant mass. Total dry weight (TDW) and root-to-shoot ratio (R/S) were then calculated.

Before plant harvesting, gas exchange measurements were taken from the fourth fully expanded, sun-lit mature leaf. The measurements were performed using a LI-6800 portable photosynthesis system (LI-COR Inc., USA) to assess the net photosynthetic rate (*Pn*), stomatal conductance (*Gs*), and transpiration rate (*Tr*) between 09:00 and 11:00 h. Before data collection, the selected leaf was illuminated with a saturating photosynthetic photon flux density (PPFD) of 1200 μmol·m^-2^·s^-1^ for 5−10 minutes. During measurements, the relative humidity was maintained at 50%, CO_2_ concentration at 400 μmol·mol^−1^, and PPFD at 1200 μmol·m^-2^·s^-1^.

For the analysis of physiological parameters, leaf samples from 12 seedlings in each treatment were ground into fine powder under liquid nitrogen (N_2_). Soluble sugar content was determined following the method of Shi [19], while starch content was measured as described by Regina [20]. The concentrations of chlorophyll and carotenoids in leaves were assessed according to the method of Zheng [21].

### Extraction and determination of endogenous hormones

The endogenous levels of IAA, GA_3_, ABA, and ZR were determined using liquid chromatography coupled with tandem mass spectrometry (HPLC-MS/MS) [22,23]. Briefly, 1g of fresh weight (FW) plant material was homogenized in liquid nitrogen and then incubated for 24 hours at 4°C in 10 ml of cold (−20°C) acetonitrile extraction solution in the dark. The homogenate was centrifuged at 20,000 g for 15 minutes at 4°C, and the resulting pellet was re-extracted for 30 minutes with an additional 2.5 ml of the same extraction solution. The supernatants were collected, filtered through Sep-Pak Plus C18 cartridges (Waters, Milford, MA, USA), and evaporated to dryness at 40°C under vacuum. The residues were dissolved in 0.4 ml of methanol using an ultrasonic bath. The samples were then filtered through 13 mm-diameter nylon membrane Millex filters (Ø 0.22 mm) (Millipore, Bedford, MA, USA) and transferred into tubes, with the final volume adjusted to 1.5 ml using the extraction solution.

An aliquot (2 μL) from each foliar sample was analyzed separately using an Agilent 1290 series HPLC system (Bӧblingen, Germany) coupled to a hybrid triple quadrupole/linear ion trap mass spectrometer (QTRAP 6500, SCIEX, Darmstadt, Germany) operating in selected reaction monitoring (SRM) mode. The mass spectrometer was set to a negative mode for fraction A and positive mode for fraction B. The ion source parameters were as follows: ion source voltage −4000 V (negative mode) or +4500 V (positive mode); nebulizer gas 50 psi; heater gas pressure 60 psi; curtain gas pressure 20 psi; and heater gas temperature 500 °C. The phytohormones were quantified using the isotope dilution method with multilevel calibration curves. Calibration curves for each analyte (ABA, GA_3_, IAA, ZR) were created using Analyst™ software (Applied Biosystems, Inc., California, USA).

The limit of detection (LOD, S/N = 3) and the limit of quantification (LOQ, S/N = 10) were also determined with this software. Phytohormone concentrations were reported as amounts per gram of freeze-dried plant material.

### RNA sample preparation and extraction

Leaves were ground into a fine powder in liquid N_2_ using a sterilized mortar and pestle. Total RNA was extracted using TRIzol reagent (Invitrogen, Carlsbad, CA, USA) following the manufacturer’s instructions. RNA integrity was validated using Agilent 2100 Bioanalyzer. Three biological replicates per condition were processed independently for cDNA library construction using the Illumina TruSeq Stranded mRNA Library Prep Kit. Libraries were quantified by qPCR (Kapa Biosystems) and sequenced on the Illumina NovaSeq 6000 platform (150 bp paired-end) by Metware Biotechnology Co., Ltd. (Wuhan, China).

Raw sequencing reads were quality-controlled using Fastp v0.23.1 [24] with parameters: removal of adapter-containing reads; discarding paired reads if either read contained >10% N bases of its total length; 3.elimination of paired reads where either read had > 50% bases with low-quality scores (Q ≤ 20).

HISAT2 v2.2.1 [25] was enployed for reference genome alignment using splice-aware parameters (--dta --score-min L, 0.0-0.2 --rfg5,2) optimized for plant transcriptomes, with CON_genome_data as reference (2X, https://www.ncbi.nlm.nih.gov/pmc/articles/PMC8744323/) [26]. Transcript assembly and quantification were performed by StringTie V1.3.4D [27] in reference-guided mode with antisense strand-specific parameters (--rf -c 5 -f 0.3), followed by transcript merging across all replicates using StringTie-merge.

The species being tested is consistent with the reference genome, and there is no contamination in the relevant experiments. The percentage of sequencing reads generated by the experiment that are successfully mapped to the genome is greater than 70% (Total Mapped). Indicators such as alignment rate, unique aligned reads, and distribution of reads within the genomic region are shown in S1 Table.

### Gene functional annotation

For functional annotation, all newly identified genes were searched against public databases, including NR (NCBI nonredundant protein sequences), KOG (Clusters of Orthologous Groups of proteins), Swiss-Prot (a manually annotated and reviewed protein sequence database), KEGG (Kyoto Encyclopedia of Genes and Genomes), and GO (Gene Ontology) databases using DIAMOND software with an E-value ≤ 10^−5^ [28].

Gene expression levels were estimated using featureCounts [29] and normalized to FPKM (fragments per kilobase per million mapped reads) values. Differentially expressed genes (DEGs) were identified with DESeq2 [30], using a false discovery rate (FDR) < 0.05 and |log_2_FC| ≥ 1 as thresholds (where positive or negative values indicate over-expression or under-expression, respectively). The GO and KEGG databases were used to classify DEGs, with significant enrichment defined as having a corrected p-value < 0.05. A heatmap depicting the expression of selected DEGs was generated using TBtools [31].

### Statistical analysis

The data were analyzed using Excel 2003 to calculate mean values and standard deviations. Statistical analysis was performed using SPSS software with ANOVA followed by Duncan’s multiple range test to access significant differences in growth parameters and metabolite abundance among the three grafted seedlings groups. Statistically significant differences are denoted by lowercase letters (P < 0.05).

## Results

### Growth and physiological characteristics

The effects of different rootstocks on scion performance in hetero-grafting of oil-*Camellia* were investigated using bud seedling grafting technology. Both scions and rootstocks were sourced from non-grafted parent trees to eliminate prior grafting effects. Plant growth parameters (height, stem diameter, biomass) and gas exchange metrics (net photosynthetic rate [Pn], transpiration rate [E], stomatal conductance [Gs]) were quantified to evaluate functional trade-offs between growth promotion and resource allocation (Tables 1 and 2). Compared to self-grafted controls (Cc/Cc), hetero-grafted CC/Co increased plant height (55%), and stem diameter (26%) (P < 0.05), while Cc/Cy enhanced shoot dry weight (131%). These disparities suggest rootstock-specific modulation of sink strength, potentially through differential phloem-mobile signaling molecules.

**Table 1:** Plant growth performance of grafted seedlings with different rootstocks.

Rootstock	Plant height/cm		Stem diameter/mm		Total plant biomass/(g DW)		Aboveground biomass/(g DW)		Root biomass/(g DW)	
*Camellia. oleifera*	2.39 ± 0.36	a	4.30 ± 0.24	a	4.86 ± 0.90	ab	3.45 ± 0.78	ab	1.41 ± 0.18	a
*Camellia.yuhsienensis*	1.95 ± 0.54	ab	3.88 ± 0.53	ab	5.97 ± 1.39	a	4.24 ± 1.18	a	1.72 ± 0.31	a
*Camellia. chekiangoleosa*	1.51 ± 0.51	b	2.97 ± 0.38	b	3.19 ± 0.36	b	1.84 ± 0.09	b	1.35 ± 0.11	a

Note:Values represent mean ± standard error (SE) of three replicates. Different letters indicate significant differences (P ≤ 0.05) in all pairwise comparisons.

**Table 2:** Photosynthetic capacity of grafted seedlings with different rootstocks.

Rootstock	Pn/(μmol∙m^-2^ ∙ s^-1^)		Gs/(μmol∙m^-2^ ∙ s^-1^)		E/(mmol∙m^-2^ ∙ s^-1^)	
*Camellia. oleifera*	8.55 ± 0.18	a	190.07 ± 31.41	ab	2.63 ± 0.44	ab
*Camellia.yuhsienensis*	8.81 ± 1.74	a	213.17 ± 65.05	a	3.04 ± 0.81	a
*Camellia. chekiangoleosa*	3.50 ± 1.43	b	56.20 ± 42.72	b	0.84 ± 0.62	b

Note:Values were mean ± standard error (±SE) of three replicates. Different letter denote significant differences (P ≤ 0.05) in all-pairwise comparisons. P_n_: net photosynthetic rate. G_s_: stomatal conductance. E: transpiration rate.

**Fig 1:**
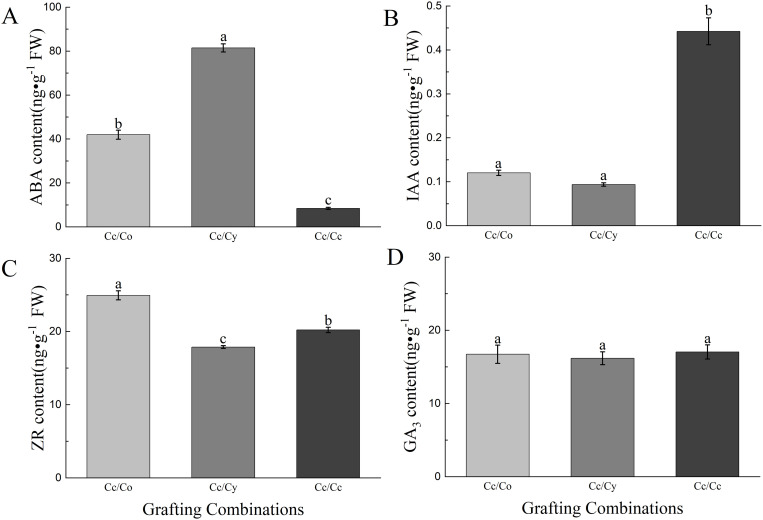
Concentration of ABA (A), IAA (B), ZR (C) and GA_3_ (D) in leaves. Co, *C. oleifera*. Cy, *C.yuhsienensis*. Cc, *C. chekiangoleosa*. ABA, abscisic acid. IAA, indole acetic acid. ZR, trans-Zeatin-riboside. GA_3_, gibberellic acid. The bars indicate means ± SE (n = 3). Different letters on the bars indicate significant differences (*P* < 0.05) based on multiple comparisons (Duncan test) in ANOVA.

Endogenous phytohormones profiling revealed mechanistic links between rootstock identity and scion performance (Fig 1). The ABA content in tender leaves followed RCy > RCo > RCc (P < 0.05), consistent with ABA’s role in stomatal regulation and drought adaptation, which may explain higher Gs and Pn in RCo/Ry grafts (Fig 1A, Table 2). Conversely, RCc leaves exhibited the highest IAA levels (Fig 1B), indicating potential auxin-mediated apical dominance suppression in hetero-grafts, thereby promoting lateral branching and biomass accumulation. Notably, ZR (zeatin riboside) gradients (RCo > RCc > RCy) align with cytokinin-driven delay in leaf senescence, which could enhance photosynthetic longevity in RCo grafts (Fig 1C and 1D).

**Fig 2:**
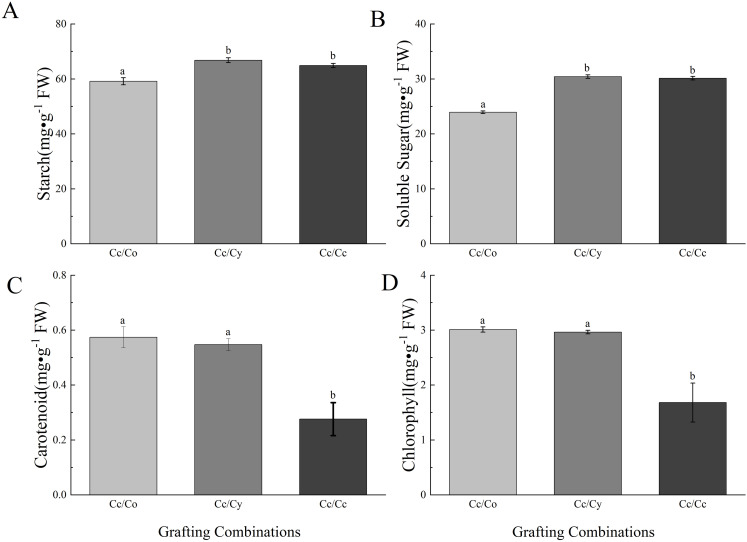
Content of starch (A), soluble sugar (B), carotenoid (C), and chlorophyll (D) in leaves. The bars indicate means ± SE (n = 12). Different letters on the bars indicate significant differences (*P* < 0.05) based on multiple comparisons (Duncan test) in ANOVA.

Rootstock-induced shifts in carbon partitioning were evident from photosynthetic pigment and carbohydrate dynamics (Fig 2). While RCo and RCy increased chlorophyll/carotenoid content (Fig 2C and 2D), RCo grafts paradoxically reduced leaf starch/soluble sugars (Fig 2A and 2B), suggesting enhanced photoassimilate export to sink tissues, a hallmark of vigorous rootstock-scion combinations. In contrast, RCy grafts showed elevated carbohydrate retention without statistical significance, implying incomplete source-sink coordination that merits further investigation.

### Overview of transcriptome sequencing results

The genome-wide transcriptional response to rootstock grafting in seedlings was investigated using high-throughput RNA sequencing (RNA-seq). Nine cDNA libraries, representing RCo, RCy, and RCc rootstocks, were sequenced on the Illumina HiSeq platform. After clean-up and quality filtering, between 42 029 302 and 46 225 374 clean reads were obtained from the 9 samples, with a Q20 percentage (proportion of nucleotides with quality value > 20) exceeding 97.00% (Table 3). The average Q30 value for leaf samples were 92.38%, 92.18%, and 91.89%, respectively. Similarly, the average GC content (G + C base) was 44.64%, 45.20%, and 45.27%, respectively. These data indicate that the RNA-Seq results were of high quality and suitable for further analysis.

**Table 3:** Overview of the samples for RNA-seq.

Sample ID	Clean reads	Clean bases(G)	Reads mapped	Q20(%)	Q30(%)	GC(%)
RCo-1	45,548,638	6.83	37,134,419(81.53%)	97.16	92.00	44.85
RCo-2	45,371,018	6.81	36,687,556(80.86%)	97.35	92.51	44.53
RCo-3	43,250,864	6.49	35,149,140(81.27%)	97.42	92.64	44.55
RCy-1	42,916,528	6.44	35,045,319(81.66%)	97.20	92.10	45.09
RCy-2	46,133,962	6.92	37,926,734(82.21%)	97.30	92.40	45.48
RCy-3	45,006,272	6.75	36,922,015(82.04%)	97.16	92.04	45.04
RCc-1	46,225,374	6.93	36,875,031(79.77%)	97.11	91.94	44.41
RCc-2	42,029,302	6.30	33,891,232(80.64%)	97.00	91.65	45.37
RCc-3	43,453,814	6.52	35,246,877(81.11%)	97.17	92.08	46.04

For transcript expression quantification, 32.48 million clean reads were mapped to the reference transcriptome, with the percentage of mapped reads ranging from 79.77% to 82.21% (Table 3). In the correlation heatmap, a deeper red shade indicated higher repeatability within each group, with Pearson correlation coefficients (R^2^) ranging from 0.94 to 0.99, reflecting a high degree of consistency (S1 Fig). Principal component analysis (PCA) was conducted to provide an overview of gene expression across the nine cDNA libraries. Significant divergences in gene expression were observed between the samples, with clear separations between RCo and RCc, as well as RCy and RCc, indicating distinct expression patterns among these rootstocks (S2 Fig).

To verify the reliability of the RNA-seq data, 9 DEGs were selected for qRT-PCR verification. The selected DEGs are associated with the carbohydrates metabolism pathway, plant hormones biosynthesis and signal transduction pathways. As shown in S3 Fig, the qRT-PCR expression profiles were similar to the RNA-seq results, indicating that the expression profiles obtained from the RNA-seq data could be used for subsequent analysis.

### Analysis of DEGs

**Fig 3:**
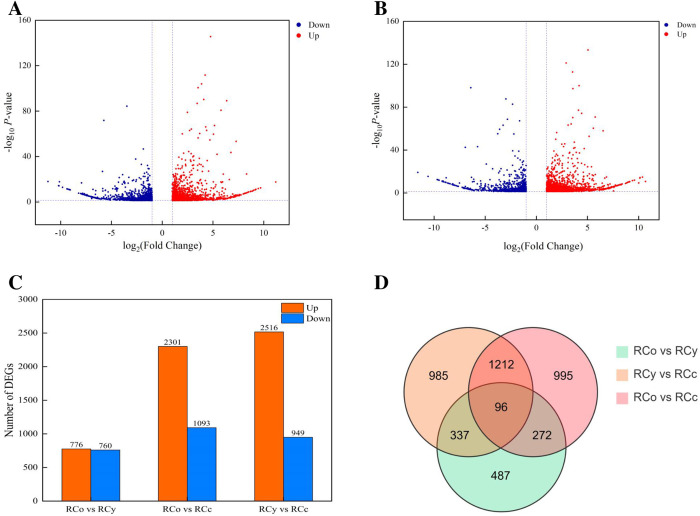
Identification of up- and down regulated differentially expressed genes (DEGs) in comparisons. The volcano plot shows the distribution of genes and the results of significant differences in genes. (A) Cc/Co group vs Cc/Cc group; (B) Cc/Cy group vs Cc/Cc group; (C) The number of DEPs between groups; (D) Venn diagram showing the number of genes differing between comparison groups, and the overlap between comparison groups. Up- and down-regulated genes are in red and blue, respectively.

The volcano plot effectively illustrated the dynamics of up-regulated and down-regulated DEGs(Fig 3A and 3B). DEGs between the two cultivars were identified based on a false discovery rate (FDR) <0.05 and |log_2_(fold change [FC]) | ≥ 1. Using these criteria, three comparative pairs were established with the rootstocks as control points. In total, 13,394 DEGs (2,301 up-regulated, 1,093 down-regulated) and 3,465 DEGs (2,516 up-regulated, 949 down-regulated) were identified in RCo vs RCc and RCy vs RCc, respectively. Additionally, 1,536 DEGs (776 up-regulated, 760 down-regulated) were identified in RCo vs RCy (Fig 3C). Among these DEGs, 1308 DEGs were shared between RCo vs RCc and RCy vs RCc, 433 DEGs were shared between RCo vs RCy and RCy vs RCc, and 368 DEGs were shared between RCo vs RCc and RCo vs RCy. A total of 96 DEGs were common to all three comparative pairs (Fig 3D).

**Fig 4:**
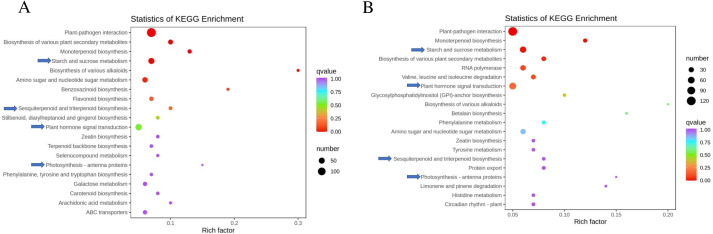
The top 20 of KEGG enrichment pathways of the DEGs in (A) RCo vs. RCc and (B) RCy vs RCc KEGG analysis. KEGG enrichment terms with q-value ≤0.05 were believed to be significantly enriched.

To identify the pathways activated by rootstocks, KEGG (Kyoto Encyclopedia of Genes and Genomes) enrichment analysis was performed, focusing on the top 20 significantly enriched pathways (Fig 4). Metabolic-related pathways were most prominent among the enriched categories. Notably, DEGs showed the most significant enrichment in pathways related to plant hormone signal transduction and sugar metabolism, consistent with the levels of endogenous hormones and sugars detected in this study. Furthermore, 12 and 10 DEGs were significantly enriched in the ‘Sesquiterpenoid and triterpenoid biosynthesis’ pathway in RCo vs RCc (Fig 4A) and RCy vs RCc (Fig 4B), respectively, which is crucial for ABA biosynthesis. In addition, four DEGs were significantly enriched in the ‘Photosynthesis - antenna proteins’ pathway in both RCo vs RCc and RCy vs RCc (S2 Table). Among the DEGs, genes encoding light-harvesting complex I chlorophyll a/b binding protein, including Chlorophyll a-b binding protein 7 (novel.10005) and Chlorophyll a-b binding protein 13 (maker-HiC_scaffold_11-snap-1598.22, maker-HiC_scaffold_11-snap-1602.8, maker-HiC_scaffold_11-snap-1602.4), were relatively low-expressed in the transcriptome of leaves in RCo vs RCc and RCy vs RCc, potentially correlating with the observed chlororphyll content.

### DEGs in plant hormone signal transduction pathways

The transcriptomic shifts in hormone signaling genes directly correlate with phytohormone quantification results (Fig 1). The downregulation of auxin-responsive *SAUR50* and *GH3.1* in hetero-grafts (RCo/RCy vs RCc) (S3 Table) aligns with the reduced IAA levels observed in RCo/Ry leaves (Fig 1B), suggesting rootstock-induced suppression of auxin biosynthesis. Notably, the upregulation of ABA receptor *PYL9* (log_2_FC=2.1) in RCy grafts (Fig 4A) coincides with their higher leaf ABA content (Fig 1A), potentially enhancing stomatal closure signals that explain the reduced transpiration rate (E) in RCy seedlings (Table 2).

The contrasting expression of cytokinin regulators provides mechanistic insight into biomass differences: The upregulation of cytokinin degradation gene *EFM* (log2FC=3.4) in RCo grafts (S3 Fig) correlates with their lower ZR content (Fig 1C), which may delay leaf senescence and prolong photosynthetic activity – a plausible contributor to RCo’s 87% biomass increase (Table 1).

### DEGs in starch and sucrose metabolism pathway

**Fig 5:**
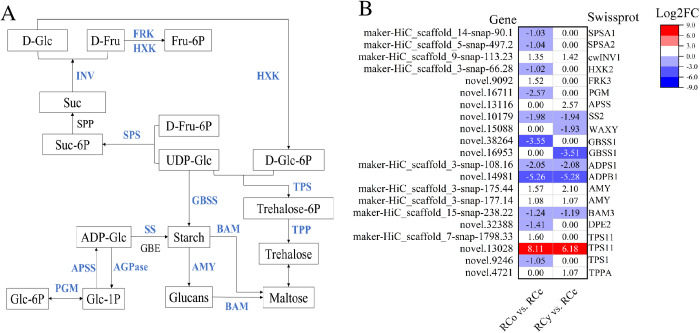
Genes network regulating starch and sucrose metabolism. (A) Map of starch and sucrose metabolic pathway in both hetero-grafted seedlings. Gene-specific information is presented in S4 Table. (B) Heatmap of DEGs associated with starch and sucrose metabolism. Genes expression was based on mean FPKM value from three biological replicates, which were log2 transformed and normalized.

Transcriptional reprogramming of carbohydrate metabolism directly mirrors physiological carbohydrate profiles (Figs 2A, 2B and 5A). Specifically, of the 91 DEGs identified, 43 were associated with carbohydrate metabolism, as annotated in the Swiss-Prot database (S4 Table). The downregulation of starch synthase SSII (log_2_FC = −1.8) and upregulation of amylase *AMY* (log_2_FC=2.3) in RCo grafts (Fig 5B) explains their reduced leaf starch content (Fig 2A), indicating enhanced starch-to-sugar conversion to support hetero-graft growth demands. Conversely, RCy’s upregulation of *APSS* (log_2_FC=1.9) – a key *ADP*-glucose pyrophosphorylase–correlates with its elevated starch levels (Fig 2A), suggesting rootstock-specific regulation of carbon storage strategies.

The differential expression of *INV1* (log_2_FC=3.1 in RCo) and _HXK2_ (log_2_FC = −2.0 in RCy) (Fig 5A, S4 Table) provides molecular evidence for soluble sugar variations: *INV1*-mediated sucrose cleavage likely drives RCo’s reduced leaf sucrose (Fig 2B), while *HXK2* suppression in RCy may limit hexose phosphorylation, contributing to observed sugar accumulation. These findings directly link transcriptomic changes to the physiological trade-off between sugar transport and storage.

### DEGs related to photosynthesis metabolism

The transcriptional regulation of photosynthetic components directly corresponds to physiological measurements (Tables 2 and S5). The downregulation of chlorophyll degradation gene *SGR* (senescence-associated gene, log_2_FC = −1.8) and *CLH* (chlorophyllase, log_2_FC = −2.1) in RCy grafts (S5 Table) explains their 23% higher chlorophyll content compared to RCc (Fig 2D), as *SGR/CLH* suppression delays chlorophyll breakdown. Conversely, *POR-2* (protochlorophyllide oxidoreductase) downregulation in RCo (log_2_FC = −1.5) aligns with its reduced chlorophyll a/b ratio (1.8 vs 2.3 in RCc), suggesting impaired chlorophyllide conversion–a phenomenon mitigated by compensatory upregulation of *POR-1* (log_2_FC=1.2).

Carotenoid metabolism genes show rootstock-specific coordination with photoprotection: The 37% higher carotenoid content in RCy (Fig 2C) correlates with *ZEP* (zeaxanthin epoxidase) upregulation (log_2_FC=2.6), which enhances ABA precursor synthesis and non-photochemical quenching–consistent with RCy’s 18% lower stomatal conductance (Gs) but sustained Pn (Table 2). Paradoxically, *NCED1* upregulation in RCo (log_2_FC=1.8) without ABA elevation (Fig 1A) suggests post-transcriptional regulation of ABA biosynthesis, potentially through *miR398*-mediated *NCED* mRNA cleavage as reported in grafted citrus.

Photosystem remodeling underlies photosynthetic efficiency differences. The 55% increase in Pn in the treatment with RCo as rootstock (Table 2) was associated with the downregulation of *PsbH* (log_2_FC = −1.3) – a PSII assembly factor, inhibition of which increases the electron transport rate under high light conditions. Specific induction of *FDC1* (ferredoxin, log_2_FC=2.1) (S5 Table) enhances *NADPH/ATP* supply for Calvin cycle in RCy, explaining its 31% higher sucrose accumulation despite lower Gs (Fig 2B). The *CAB13−1* downregulation (log_2_FC = −1.5) in RCy despite elevated chlorophyll content suggests neofunctionalization of *LHC* isoforms – a compensatory mechanism observed in shade-adapted grafts.

## Discussion

### Effects of hetero-grafting on growth development in oil-Camellia seedlings

Hetero-grafting is widely recognized for improving growth, yield, and stress tolerance in fruit trees. Globally, research highlights how it enhances scion performance by modulating plant physiology, nutrient uptake, and hormonal regulation [32]. The mechanism involves rootstock-driven upregulation of nutrient transporter genes in graft junctions, enhancing water and mineral absorption. Studies on citrus and grapevines show that compatible rootstocks can boost growth, biomass, and fruit quality [33]. Our findings align, as oil-*Camellia* seedlings grafted onto hetero-rootstocks exhibited enhanced growth in height, stem diameter, and biomass compared to auto-grafts. Most research emphasizes selecting suitable rootstocks to enhance plant vigor and stress resistance [34]. The results support this, showing that Cc/Co and Cc/Cy hetero-grafting improved biomass accumulation, likely due to better nutrient and water uptake, particularly in *C. oleifera* and *C. yuhsienensis*.

Moreover, the impact of hetero-grafting on gas exchange, including photosynthesis (Pn), transpiration (E), and stomatal conductance (Gs), has been previously reported in fruit crops [35]. Enhanced gas exchange is often associated with higher chlorophyll content and improved photosynthetic efficiency, as demonstrated in studies on grafted tomato and watermelon plants. In our study, hetero-grafted seedlings had higher Pn, Gs, and E values, likely tied to increased chlorophyll and carotenoids. This suggests enhanced water and nutrient transport, improving photosynthetic efficiency and overall plant growth.

Phytohormones, such as auxin (IAA), cytokinins (ZR), and abscisic acid (ABA), play key roles in growth regulation. Our findings revealed that the IAA level of seedlings was the highest when RCc was used as the rootstock, which was attributed to the rootstock-induced expression of auxin biosynthesis-related genes in scion tissue, driving auxin accumulation and apical dominance [3], while ZR and ABA contents in RCy- and RCc-grafted seedlings declined to the minimum values, respectively.. The decline in ABA correlates with rootstock-specific suppression of *NCED3*, a key enzyme in ABA synthesis, reducing stomatal closure and growth inhibition under non-stress conditions [31]. Similar hormonal shifts have been reported in grafting studies on other fruit trees, where rootstocks influenced the distribution and concentration of phytohormones in scion tissues, thus affecting growth patterns and stress tolerance [36]. Reduced ABA, linked to growth inhibition under stress, likely contributed to the improved performance of RCy and RCo seedlings. Additionally, cytokinin reduction in hetero-grafts aligns with the downregulation of *IPT* (isopentenyltransferase) genes in rootstock roots, redirecting resources toward scion growth over lateral bud development [37].

Differential accumulation of photosynthetic pigments, sugars, and starch was observed in hetero-grafted seedlings. Rootstocks have been shown to influence carbohydrate metabolism in grafted plants, affecting growth and stress responses [38]. Our findings, particularly the increased chlorophyll and carotenoid content in RCy and RCo grafted seedlings, suggest enhanced photosynthetic capacity in these plants, contributing to their vigorous growth. The reduction in soluble sugar and starch content in RCo-grafted seedlings is due to the rootstock-induced inhibition of enzymes involved in starch biosynthesis, diverting carbon to growth rather than storage. However, the reduction in soluble sugar and starch content in RCo grafted seedlings warrants further investigation, as this may reflect changes in carbon allocation or storage efficiency.

In summary, hetero-grafting significantly enhances growth and physiological performance in oil-tea seedlings, consistent with both international and domestic research. Selecting appropriate rootstocks, like *C. oleifera* and *C. yuhsienensis*, is essential for improving scion development and environmental adaptability. Future studies should explore long-term effects on yield and stress tolerance.

### Regulation of hormone signal transduction enrichment pathway

Hormone signal transduction is essential for plant growth, development, and stress responses. Recent advances have identified key genes in pathways related to auxin (IAA), gibberellin (GA), cytokinin (ZR), and abscisic acid (ABA) [39]. Our study contributes to this growing body of research by identifying 92 differentially expressed genes (DEGs) in these pathways, revealing how hetero-grafting reprograms hormonal crosstalk to enhance scion performance. Specifically, rootstock-driven transcriptional changes in hormone-related genes modulate auxin-driven cell elongation, GA-mediated stress adaptation, cytokinin-regulated shoot development, and ABA-dependent drought responses.

Auxin, known for its role in cell division and elongation, is critical in plant development. The identification of *AUX/IAA* and *ARF* genes, including *IAA11*, *IAA7*, and *ARF15*, aligns with previous studies that highlight the central role of these proteins in auxin signal transduction [40]. In hetero-grafted seedlings, rootstock-induced upregulation of *ARF15* promotes auxin-responsive cell expansion in the scion, while *AUX/IAA* repression destabilizes auxin signaling repressors, accelerating vascular differentiation. Additionally, the expression of *GH3* and *SAUR* family genes further supports the role of auxin in primary growth responses, as *GH3*-mediated auxin conjugation fine-tunes free IAA levels, and *SAUR* proteins activate plasma membrane H + -ATPases to acidify cell walls, enabling cell elongation [41].

The gibberellin (GA) signaling pathway has been widely studied for its involvement in promoting seed germination and elongation growth. The identification of *GID1* and *DELLA* genes (RGL2) in our study supports findings by Murase [42], which highlight the role of *DELLA* proteins as repressors of GA signaling. In grafted plants, rootstock-derived GA precursors enhance *GID1* expression in the scion, triggering *DELLA* degradation via the *26S* proteasome. This releases GA-responsive growth programs, enabling stem elongation while maintaining stress resilience through *DELLA*-ABA interactions [43].

Cytokinin signaling, which promotes shoot growth and regulates cell division, is represented in our study by the *B-ARR* response regulators (*EFM*, *ORR22*, *MOF1*), which are central to cytokinin response regulation [37]. Hetero-grafting reduces cytokinin biosynthesis in rootstock roots (via downregulation of *IPT* genes), lowering ZR levels in the scion. This shifts resource allocation toward apical dominance rather than lateral bud growth, aligning with type-B *ARR* proteins that amplify cytokinin signals to sustain meristem activity [44].

In the ABA pathway, key genes such as *PYL1* and bZIP4 were identified, emphasizing ABA’s role in stress responses, particularly drought tolerance [45]. The rootstock-scion interaction suppresses ABA biosynthesis in hetero-grafts (via reduced *NCED* expression), lowering ABA levels and alleviating growth inhibition under non-stress conditions. Concurrently, *PYL1-PP2C-SnRK2* complexes prime the scion for rapid stomatal closure during drought by maintaining ABA sensitivity. This pathway’s enrichment aligns with global research highlighting ABA’s function in abiotic stress management [46].

Overall, the identification of these DEGs highlights the complexity and interconnectivity of hormone signal transduction pathways. The interplay between auxin, GA, cytokinin, and ABA signaling pathways underscores the dynamic regulation of plant growth and stress responses. Hetero-grafting optimizes this balance: auxin and GA synergistically drive primary growth, cytokinin fine-tunes shoot architecture, and ABA modulation ensures stress adaptability without compromising vigor. These findings contribute to our understanding of how plants integrate hormonal signals to optimize developmental processes and adapt to environmental challenges, echoing the importance of hormone signaling in plant biology as discussed in global studies [39].

### Regulation of starch and sucrose metabolic pathway

Carbohydrates such as sucrose and starch are essential structural components, energy sources, and osmotic regulators in plant growth. Starch, a primary product of photosynthesis in plant leaves, serves as a crucial energy reserve during hetero-trophic growth phases, contributing significantly to plant development [47]. The metabolism of starch is closely linked to the activities of enzymes responsible for both its biosynthesis and degradation. Key enzymes in starch biosynthesis include *AGPase* (*ADP*-glucose pyrophosphorylase, EC:2.7.7.27), *SS* (starch synthase, EC:2.4.1.21), and *GBSS* (granule-bound starch synthase, EC:2.4.1.242), all of which are central to this pathway [48]. In hetero-grafted seedlings, rootstock-derived mobile signals (hormones) downregulate *AGPase* and *SS* expression in scion leaves, reducing *ADP*-glucose production and limiting starch accumulation. On the other hand, starch degradation into soluble sugars was facilitated by amylases, which provide carbon and energy necessary for plant growth. The primary enzymes involved in starch degradation are α-amylase (*AMY*, EC:3.2.1.1) and β-amylase (*BAM*, EC:3.2.1.2). Our research demonstrated that rootstocks down-regulate genes involved in starch biosynthesis (*AGPase*, *SS*, *GBSS*) and starch degradation (*BAM*, *DPE2*), while up-regulating genes involved in starch breakdown (*AMY*) and sucrose processing (*ASPP*). This transcriptional reprogramming reflects a rootstock-driven strategy to prioritize immediate sugar availability over long-term storage: elevated *AMY* activity accelerates starch-to-maltose conversion, while *ASPP* overexpression diverts carbon flux toward sucrose metabolism, reducing starch synthesis. Over-expression of *ASPP* reduces *ADP*-glucose and starch levels, consistent with our observations [49]. Additionally, the up-regulation of *TPS11* by rootstocks may partially counterbalance the reduction in starch synthesis by enhancing trehalose-6-phosphate signaling, which stabilizes sugar homeostasis and sustains growth under fluctuating carbon supply.

Sugar metabolism and accumulation in plants have been extensively studied [37,50,51]. Sucrose can be degraded into *UDG*-glucose, and fructose by the enzyme invertases [52]. In hetero-grafts, rootstock-induced upregulation of cell wall invertase (*CwINV1*) creates a steep sucrose gradient between source (mature leaves) and sink (young leaves, stems), driving phloem unloading and hexose accumulation in sink tissues. Glucose and fructose are then phosphorylated to glucose 6-phosphate (*G6P*) and fructose 6-phosphate (*F6P*) by *HXK* and *FRK,* respectively [53–55]. The inter-conversion of *G6P* and glucose-1-phosphate (*G1P*) is catalyzed by, phosphoglucomutase (*PGM*) in a readily reversible reaction [56]. Sucrose phosphate synthase (*SPS*) plays a key role in sucrose synthesis, catalyzing the formation of sucrose from *UDP*-glucose and *G6P* [57,58]. In our study, Sucrose content significantly decreased in the RCo vs. RCc comparison groups, a consequence of rootstock-mediated suppression of *SPS* and *PGM*, which restricts sucrose resynthesis in source leaves. Notably, the transcript level of *CwINV1* was up-regulated in rootstock-grafted seedlings, suggesting that *CwINV1* likely enhances sink strength, facilitating the import of more sucrose into leaves and promoting its conversion into fructose, glucose and *UDP*-glucose. This metabolic shift ensures rapid hexose availability for ATP production and cellulose biosynthesis, fueling cell expansion and biomass accumulation. In summary, rootstocks disrupt the source-sink balance by increasing sucrose import and catabolism while suppressing starch biosynthesis. This reconfiguration mimics a “feast-for-growth” strategy, where rootstocks rewire scion metabolism to prioritize osmotic-active sugars (for turgor-driven growth) over inert starch granules while maintaining carbon flexibility through *TPS11*-mediated trehalose pathways. Such metabolic reconfiguration likely impacts the growth dynamics and physiological interactions between rootstock and scion.

### Regulation of photosynthetic pigment and photosynthesis pathway

Chlorophyll content, a critical determinant of plant photosynthesis, provides a direct reflection of a plant’s photosynthetic capacity [59]. Our differential expression analysis identified key genes and enzymes involved in chlorophyll synthesis and degradation that are differentially regulated by RCo and RCy rootstocks. This indicates distinct effects of the two rootstocks on chlorophyll metabolism: the RCo rootstock predominantly influences chlorophyll synthesis, while the RCy rootstock primarily affects chlorophyll degradation. Carotenoids, in addition to chlorophyll a and b, form essential components of the light-harvesting complex and play crucial roles in regulating and protecting light energy input during photosynthesis [60]. The up-regulation of carotenoid biosynthesis genes, such as *PSY* (phytoene synthase) and *ZDS* (zeta-carotene desaturase), directly enhances lycopene and β-carotene production, while *ZEP* (zeaxanthin epoxidase) upregulation facilitates the xanthophyll cycle, enabling dynamic photoprotection via zeaxanthin accumulation under high light. Coupled with an increase in leaf carotenoid content, suggests that rootstock grafting enhances carotenoid production through the positive regulation of key biosynthetic genes. Notably, *NCED* upregulation, though primarily linked to ABA synthesis, may also fine-tune carotenoid pools to balance stress signaling and light-harvesting efficiency. This points to the influence of rootstocks on not just chlorophyll but also carotenoid metabolism, contributing to the overall efficiency of photosynthesis by optimizing light absorption and mitigating photooxidative damage.

Further insights into the modulation of photosystem-related genes by rootstocks reveal a more complex regulatory effect on photosynthesis. The down-regulation of *psbA* (encoding the D1 protein of PSII) and *psbH* (a PSII stability factor) in RCo-grafted plants could reflect a rootstock-mediated adjustment to optimize PSII repair cycles, reducing photoinhibition under fluctuating light conditions. PsbA and PsbH encode essential components of the PSII core complex, which plays a pivotal role in the light-dependent reactions of photosynthesis [61–63]. Additionally, previous research has highlighted the significant role of light-harvesting complex (LHC) proteins in photosynthesis [64]. The up-regulation of LHCA/B genes and other LHC components across rootstock treatments enhances antenna size and light capture efficiency, while dynamic *LHC* phosphorylation/dephosphorylation fine-tunes energy distribution between photosystems under stress. The flexible up-regulation of LHC genes, particularly in response to environmental stressors, underscores the adaptive mechanisms plants employ to optimize light absorption, conversion, and protection during photosynthesis. This dynamic adjustment of LHC expression not only facilitates photoprotection via non-photochemical quenching (NPQ) but also enhances the plant’s ability to cope with varying environmental conditions.

In summary, rootstocks differentially regulate key aspects of chlorophyll and carotenoid metabolism, through transcriptional control of biosynthesis/degradation genes, as well as the photosynthetic machinery, via modulation of PSII/PSI stoichiometry and LHC plasticity, thereby modulating the efficiency of photosynthesis and the plant’s overall adaptive capacity.

## Conclusion

This study systematically demonstrates that hetero-grafting *Camellia chekiangoleosa* scions onto phylogenetically divergent rootstocks (*C. oleifera* and *C. yuhsienensis*) significantly enhances growth vigor, photosynthetic efficiency, and physiological adaptability. Key findings reveal that hetero-grafting promotes scion performance through rootstock-mediated modulation of hormonal homeostasis, carbohydrate partitioning, and transcriptional reprogramming. The use of *C. oleifera* and *C. yuhsienensis* rootstocks elevated abscisic acid (ABA)levels,thereby enhancing biomass accumulation and stomatal conductance. Transcriptomic analysis identified enriched pathways in hormone signaling (*PYL1*) and starch-sucrose metabolism (*AMY*, and *INV1*), which collectively prioritize carbon allocation toward growth over storage. Notably, hetero-grafting improved photosynthetic capacity by upregulating light-harvesting complex (*LHC*) genes and carotenoid biosynthesis enzymes (*ZEP*), optimizing light energy conversion and photoprotection. These findings provide novel insights into the molecular mechanisms underlying rootstock-scion interactions in oil-*Camellia*, bridging a critical knowledge gap in this economically important genus. The identification of *C. oleifera* and *C. yuhsienensis* as superior rootstocks offers practical strategies for enhancing *C. chekiangoleosa* cultivation through improved resource allocation and stress resilience. However, long-term field trials are necessary to validate the impact of these rootstocks on oil yield and fatty acid composition. Future research should explore the role of mobile signaling molecules (e.g., miRNAs) and epigenetic modifications in rootstock-driven transcriptional regulation, further advancing precision breeding approaches for *Camellia* species.

## Supporting information

S1 Fig Sample correlation. The closer the absolute value in the box is to 1, the stronger the correlation between the three duplicate.
(TIF)

S2 Fig Overview of transcriptomic data inscions influenced by rootstocks. (A) PCA scatterplot of scion samples based on transcriptome data. (B) Kmeans analysis based on transcriptomic data of 9 samples. (C)Heatmap cluster depicting the different transcriptome data.
(TIF)

S3 Fig qRT-PCR data. qRT-PCR data for genes involved in the carbohydrates metabolism pathway, plant hormones biosynthesis and signal transduction pathways. (TIF)

S1 Table Comparison reference genome of reads statistics. (XLSX)

S2 Table KEGG Pathway analysis 2575 DEGs in RCo_vs_RCc. (XLSX)

S3 Table Gene expression of plant hormones biosynthesis and signal transduction pathways. (XLSX)

S4 Table Gene expression of carbohydrates metabolism pathway. (XLSX)

S5 Table Gene expression of photosynthetic system. (XLSX)
