# Supplementary figures and images for: Transcriptome analysis reveals rootstock-driven effects on growth and photosynthesis in Camellia chekiangoleosa: A phenotypic and biochemical perspective
Source: PLoS One. 2025 Sep 3;20(9):e0331313. doi: 10.1371/journal.pone.0331313 (PMC12407475; doi:10.1371/journal.pone.0331313)

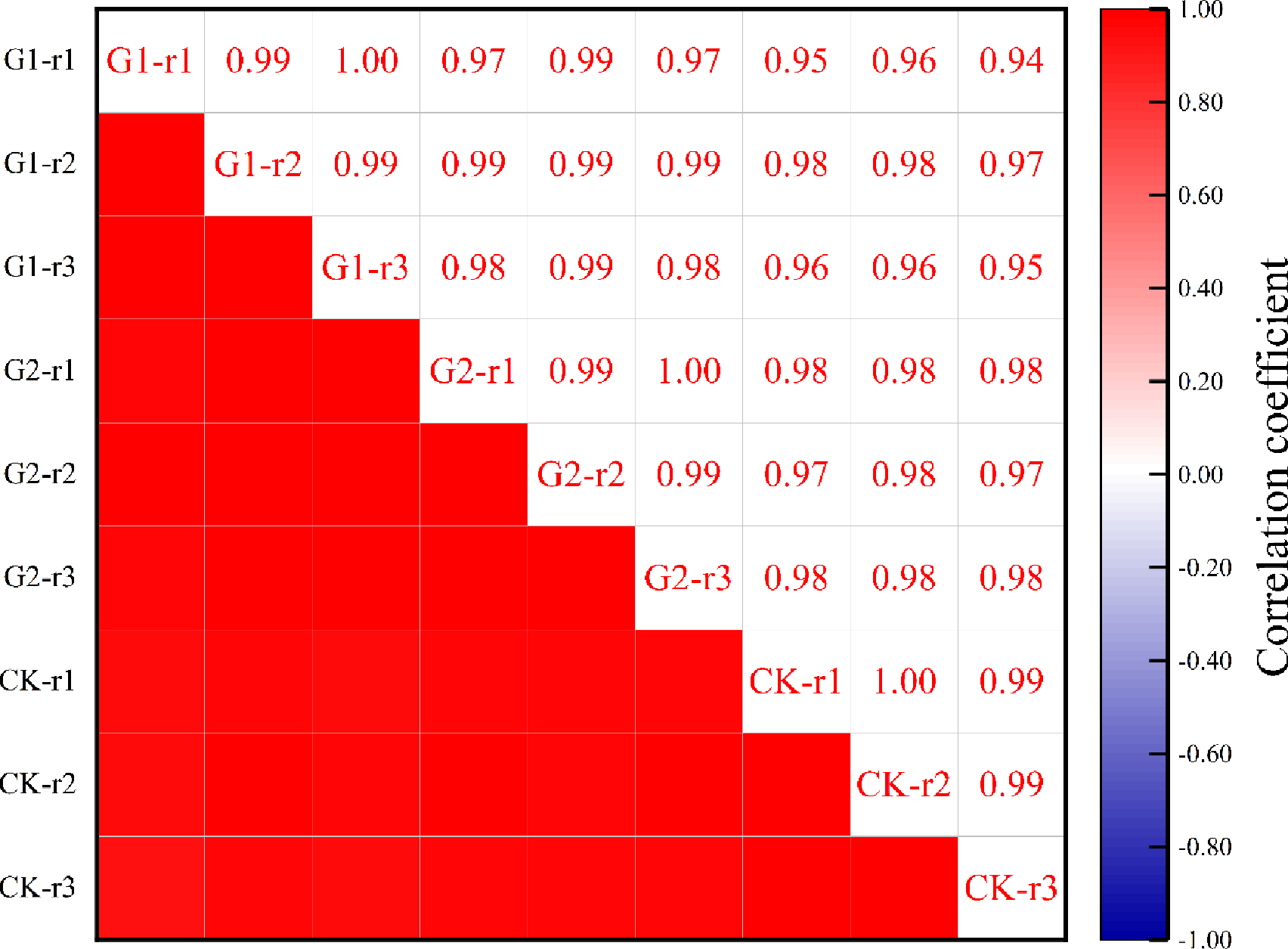

Supplement: S1 Fig — The closer the absolute value in the box is to 1, the stronger the correlation between the three duplicate. (TIF) [file pone.0331313.s001.tif]

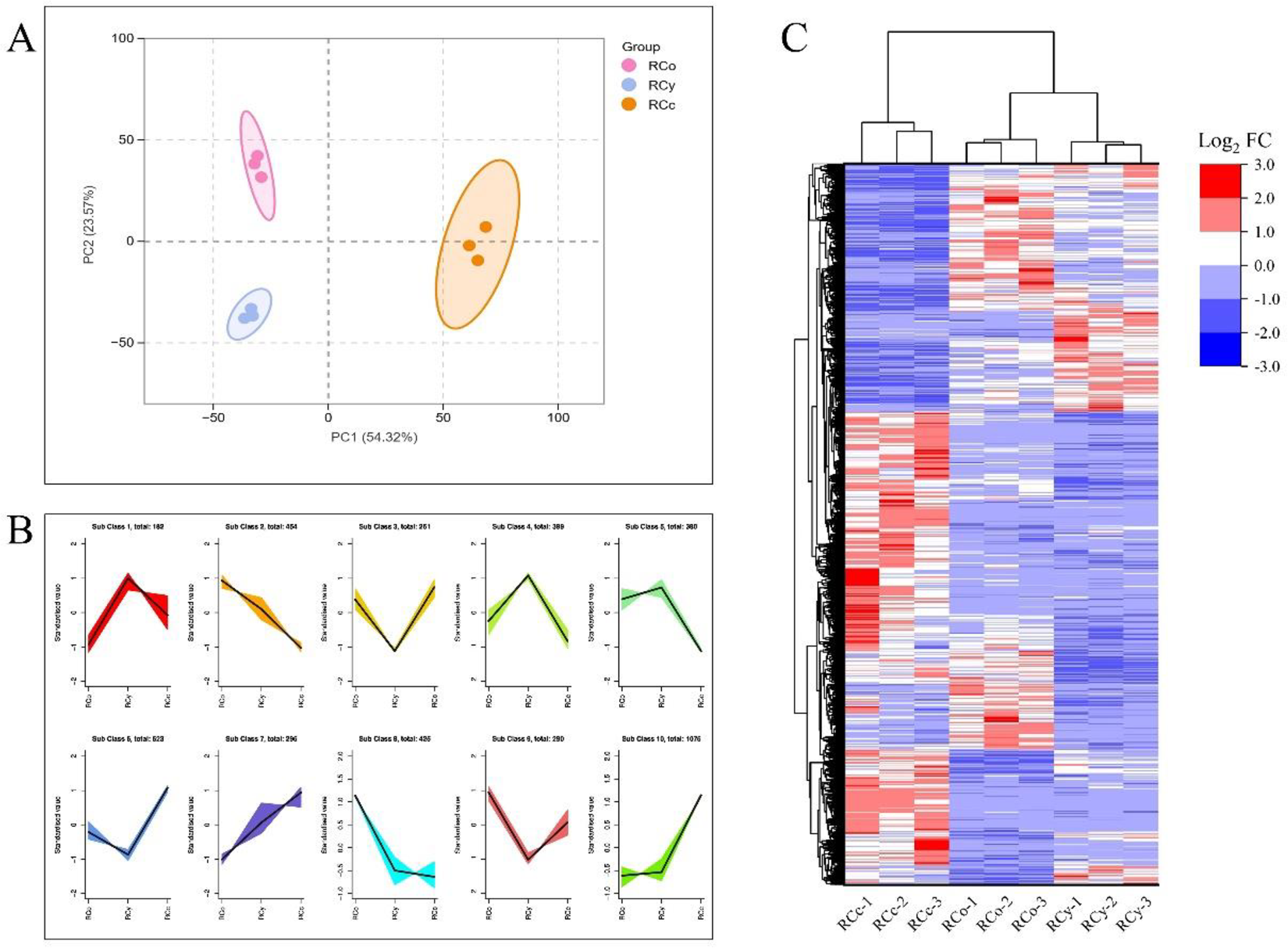

Supplement: S2 Fig — (A) PCA scatterplot of scion samples based on transcriptome data. (B) Kmeans analysis based on transcriptomic data of 9 samples. (C)Heatmap cluster depicting the different transcriptome data. (TIF) [file pone.0331313.s002.tif]

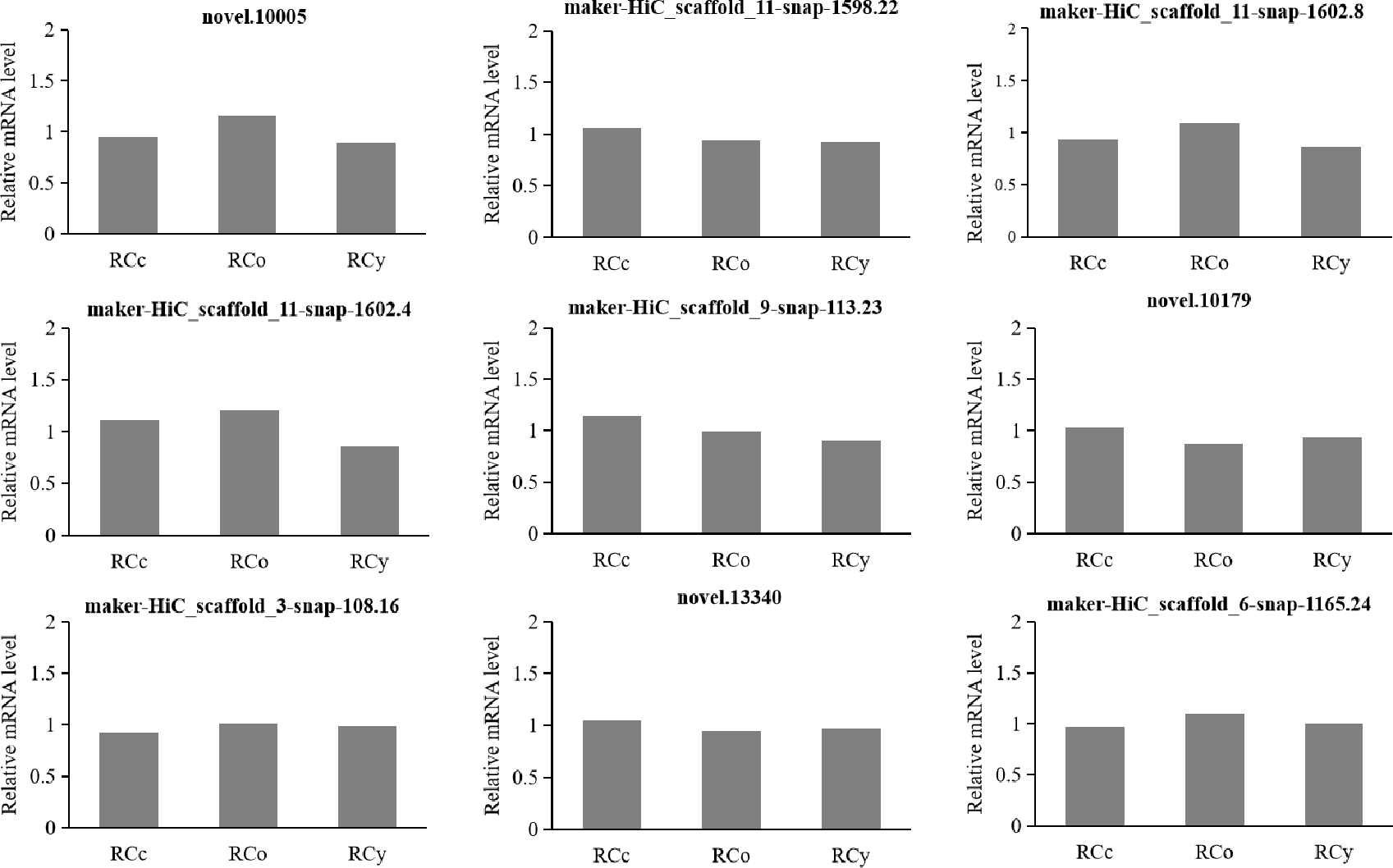

Supplement: S3 Fig — (TIF) [file pone.0331313.s003.tif]
